# Epidemiology of heart failure and long-term follow-up outcomes in a north-African population: Results from the NAtional TUnisian REgistry of Heart Failure (NATURE-HF)

**DOI:** 10.1371/journal.pone.0251658

**Published:** 2021-05-20

**Authors:** Leila Abid, Salma Charfeddine, Ikram Kammoun, Manel Ben Halima, Hedi Ben Slima, Meriem Drissa, Khadija Mzoughi, Dorra Mbarek, Leila Riahi, Saoussen Antit, Afef Ben Halima, Wejdene Ouechtati, Emna Allouche, Mehdi Mechri, Chedi Youssfi, Ali Khorchani, Kais Sammoud, Khaled Zaouia, Rami Tlili, Sana Ouali, Faten Triki, Sonia Hamdi, Selim Boudich, Marwa Chebbi, Mouna Hentati, Amani Farah, Habib Triki, H. Ghardallou, H. Radoui, Sofien Zayed, F. Azaiez, Fadoua Omri, Akram Zouari, Zine Ben Ali, A. Najjar, Houssem Thabet, Mouna Chaker, Samar Mohammed, Abdelhamid Ben Jmaa, Haithem Tangour, Yassine Kammoun, Mahmoud Cheikh Bouhlel, S. Azeiz, R. Gtaief, S. Mashki, Aymen Amri, Hela Naanea, Raoudha Othmani, Iheb Chahbani, Houcine Zargouni, Syrine Abid, Mokded Ayari, Ines Ben Ameur, Ali Guesmi, Nejeh Ben Halima, Habib Haouala, Wafa Fehri, Essia Boughzela, Lilia Zakhama, Soraya Ben Youssef, Wided Nasraoui, Rachid Boujneh, Nedia Barakett, Sondos Kraiem, Hbiba Drissa, Ali Ben Khalfalah, Habib Gamra, Salem Kachboura, Yosra Majdoub, Elifa Kanoun, Faiez Zannad, Sami Milouchi, Alexandre Mebaza, Samir Kammoun, Sami Mourali, Karima Hezbri, Faouzi Addad

**Affiliations:** 1 Tunisian Society of Cardiology and Cardiac Surgery, Tunis, Tunisia; 2 Department of cardiology, Hedi Chaker Hospital, Sfax, Tunisia; 3 Department of cardiology, Abderrahman Mami Hospital, Ariana, Tunisia; 4 Department of cardiology, La Rabta Hospital, Tunis, Tunisia; 5 Department of cardiology, Menzel Bourguiba Hospital, Bizerte, Tunisia; 6 Department of cardiology, Habib Thameur Hospital, Tunis, Tunisia; 7 Department of cardiology, Mongi Slim Hospital, La Marsa, Tunisia; 8 Department of cardiology, Hospital of the Internal Security Forces, La Marsa, Tunisia; 9 Department of cardiology, Charles Nicolle Hospital, Tunis, Tunisia; 10 Department of cardiology, Habib Bourguiba Hospital, Medenine, Tunisia; 11 Department of cardiology, Habib Bougatfa Hospital, Bizerte, Tunisia; 12 Department of cardiology, Principal Military Hospital, Tunis, Tunisia; 13 Department of cardiology, Fattouma Bourguiba Hospital, Monastir, Tunisia; 14 Department of cardiology, Mohamed Taher Al Maamouri Hospial, Nabeul, Tunisia; 15 Department of cardiology, Sahloul Hospial, Sousse, Tunisia; 16 Department of cardiology, Mohamed Ben Sassi Hospital, Gabes, Tunisia; 17 Department of cardiology, Ibn El Jazzar Hospital, Kairouan, Tunisia; 18 Department of cardiology, Kasserine Hospital, Kasserine, Tunisia; 19 Department of community Medicine, Hedi Chaker Hospital, Sfax, Tunisia; 20 Nuclear Biotechnology and Technology Laboratory - National Center for Nuclear Science and Technology of Sidi Thabet, Ariana, Tunisia; 21 Department of cardiology, Nancy Regional and University Hospital Center, Nancy, France; 22 Department of Cardiology, Lariboisière Hospital, Paris, France; 23 Molecular and Genomic Bacteriology Laboratory, National Institute of Applied Sciences and Technology, Tunis, Tunisia; University of Pittsburgh Medical Center, UNITED STATES

## Abstract

The NATURE-HF registry was aimed to describe clinical epidemiology and 1-year outcomes of outpatients and inpatients with heart failure (HF). This is a prospective, multicenter, observational survey conducted in Tunisian Cardiology centers. A total of 2040 patients were included in the study. Of these, 1632 (80%) were outpatients with chronic HF (CHF). The mean hospital stay was 8.7 ± 8.2 days. The mortality rate during the initial hospitalization event for AHF was 7.4%. The all-cause 1-year mortality rate was 22.8% among AHF patients and 10.6% among CHF patients. Among CHF patients, the older age, diabetes, anemia, reduced EF, ischemic etiology, residual congestion and the absence of ACEI/ ARBs treatment were independent predictors of 1-year cumulative rates of rehospitalization and mortality. The female sex and the functional status were independent predictors of 1-year all-cause mortality and rehospitalization in AHF patients. This study confirmed that acute HF is still associated with a poor prognosis, while the mid-term outcomes in patients with chronic HF seems to be improved. Some differences across countries may be due to different clinical characteristics and differences in healthcare systems.

## Introduction

It is estimated that 26 million people worldwide suffer from heart failure (HF) [1]. The prevalence of HF (HF) is increasing [1]. In the United States and Europe, HF is responsible for a large proportion of morbi-mortality [2–4]. In Tunisia and north-african countries, HF is a public health problem considering its current frequency and this is mainly linked to an aging of the Tunisian population (Africa’s oldest population, with the highest life expectancy of the continent) and an increase in coronary and hypertensive patients. However, there is no extensive data available on demographic characteristics, prognosis and quality of care of patients with HF in Tunisia (nor in North Africa). The data of the European and United States populations cannot be extrapolated to the Tunisian population. The aims of the present study were to determine the epidemiological profile of acute and chronic HF patients, to assess the 1-year outcomes (death and rehospitalization) of patients with HF, and to identify prognostic predictors of these outcomes.

## Materials and methods

### Study design and patient’s enrollment

The NATURE- HF registry was a national, Tunisian, observational, longitudinal, prospective and multicentric registry carried out on a follow-up period of 13 months: 01 month of inclusion and 12 months of follow-up. The protocol of the NATURE HF registry has been approved by the Tunisian Society of Cardiology and Cardiovascular Surgery. The NATURE HF study has been submitted to ClinicalTrials.gov and registered under the identifier NCT03262675. An ethical approval letter has been obtained from the ethic committee of the Abderrahmen Mami Pneumology and Phthisiology Hospital. Any selected patient will be introduced in the study and its explicit agreement will be solicited by signing an informed consent form. No data were collected before the patient received detailed information and gave signed informed consent. We included all outpatients with chronic HF (CHF) and those hospitalized for acute HF (AHF) de novo or not. Any violation of the study protocol will be exposed to the Steering Committee which will decide on the exclusion of the patient in question. The selection of patients eligible for inclusion and non-inclusion criteria will be made at the cardiology consultation level or during cardiology or emergencies hospitalizations. A total of 250 Cardiologists (public sector and liberal sector) participate in the inclusion. Patient inclusion will occur consecutively until the end of the inclusion period. The inclusion began on October 02, 2017 for a duration of 01 months. A Regular follow-up was done up to 12 months after inclusion. Given the observational nature of the NATURE-HF study, no specific treatment or intervention is planned in the management of heart failure. Patients should be cared for according to the usual medical habits.

The diagnosis of heart failure is at the discretion of investigator. The main non-inclusion criteria are the estimated life expectancy <12 months for extra-cardiac pathology, isolated right heart failure, pregnant woman, end-stage or severe renal insufficiency with creatinine clearance <15ml / min, hemodialysis patient, cardiac surgery scheduled within 3 months and congenital heart disease.

The data were collected via the DACIMA Clinical Suite^®^ web interface. The platform complies with international standards: FDA 21 CFR part 11(Food and Drug Administration 21 Code of Federal Regulations part 11), HIPPA (Health Insurance Portability and Accountability Act), ICH (International Conference on Harmonisation), MedDRA (Medical Dictionary for Regulatory Activities) and "Health Canada" and Tunisian regulations.

#### Statistical analysis

Qualitative variables were expressed as percentages. For quantitative variables, we checked the normality of the distribution by the Kolmogorov-Smirnov test and the Shapiro-Wilk test. An estimate of the means with their standard deviations and of the median with min and max was thus carried out. The baseline characteristics and type of treatments were also reported. The plots of Kaplan–Meier curves for time to all-cause death and time to all-cause death or HF hospitalization were performed. The associations between the variables were studied using hypothesis tests. The comparison between two qualitative variables was carried out by the Pearson “chi2” test when the conditions were verified otherwise the exact Fisher test was used. The Student test was used for the comparison of two means when the distribution is Gaussian and by the non-parametric U test of Mann-Whitney when the distribution is not Gaussian. In addition, these plots were divided into outpatients with CHF and inpatients with AHF. Plots of cumulative incidence of HF hospitalization considering competing risks of death in the two groups were presented.

The factors associated with 1-year death and 1-year death and rehospitalization were studied by calculating unadjusted (in univariate analysis) and adjusted (ORa) Odds Ratio after multivariate analysis using binary logistic regression. We retained a risk of error of 20% to include the indicator variables in the multivariate analysis. Age, systolic blood pressure and ejection fraction (EF) were considered as continuous variables while the remaining were considered as categorical variables. A P-value <0.05 was considered statistically significant.

## Results and discussion

A total of 2040 patients were included in the study. Of these, 408 (20%) were inpatients hospitalized with a diagnosis of acute HF (AHF) and 1632 (80%) were outpatients with chronic HF (CHF). The mean hospital stay was 8.7 ± 8.2 days [2; 55].

### Baseline characteristics

The baseline characteristics of the study population are reported in «Table 1». A larger percentage of AHF patients had reduced EF, defined as EF <40% (53.9% vs. 44.9%). The mean age was 63.56 ±12.6 years old and 29.1% were female. Around the half of HF patients had an ischemic etiology. Common co-morbidities [atrial fibrillation, diabetes mellitus, anemia, renal dysfunction and chronic obstructive pulmonary disease (COPD)] were more frequent among AHF patients.

**Table 1:** Baseline characteristics of the study population.

	Total population (n = 2040)	Acute HF (n = 408)	Chronic HF (n = 1632)	p-value
Age (years)				
Mean ± SD	63.56 ±12.6	63.59 ±12.9	63.54 ±12.5	0.89
Median [IQR]	64 [19–97]	64 [24–91]	64 [19–97]	
≥ 75 years (%)	20.7	24	19.8	0.06
Females (%)	29.1	28.7	29.3	0.85
Diabetes (%)	35.8	41.2	34.5	0.01
Hypertension (%)	40.4	42.2	40	0.42
Smoking (%)	26.2	21.7	27.7	0.11
COPD (%)	6.8	10.5	5.8	0.001
Coronary heart disease (%)	46.2	41.7	47.4	0.03
NYHA III—IV (%)	34.4	65.9	26.5	0.000
SBP (mmHg)				
Mean ± SD	123.37± 24.8	122.28± 29.0	123.95± 23.8	0.42
Median [IQR]	120 [90–240]	120 [100–240]	120 [90–230]	
≤ 110 mmHg (%)	0.5	0.6	0.5	0.68
Heart rate (bpm)				
Mean ± SD	80.17± 17.5	88.10± 22.3	78.24± 15.6	0.000
Median [IQR]	77 [30–160]	84 [39–152]	75 [30–161]	
≥ 70 bpm (%)	77.1	85.1	75.2	0.000
ECG abnormalities				
Atrial fibrillation (%)	15.9	21.3	14.5	0.000
LBBB (%)	9	10	8.8	
QRS duration				
>150 msec (%)	3.8	5.4	3.4	0.002
Ejection fraction (%)				
Mean ± SD	38.42 ± 10.5	36.96 ± 11.4	38.73 ± 10.2	0.005
Median [IQR]	40 [15–63]	37 [12–65]	40 [14–87]	
< 40%	46.5	53.9	44.9	0.001
40–50%	45.8	36.5	48	
>50%	7.7	9.7	7.1	
Mitral regurgitation (%)	22.7	24.5	22.3	0.39
Renal dysfunction (%)	38.4	46.5	36.6	0.03
Anemia (%)	7.7	13.5	6.3	0.000

### Follow-up

**Fig 1:**
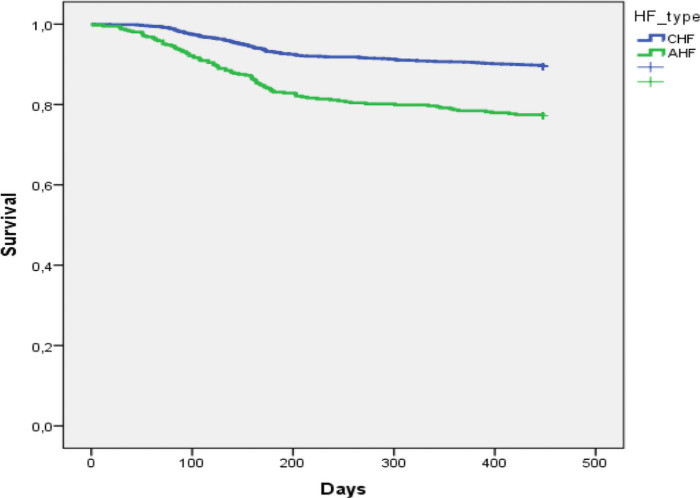
Kaplan–Meier curves for all-cause mortality in acute heart failure and chronic heart failure patients.

**Fig 2:**
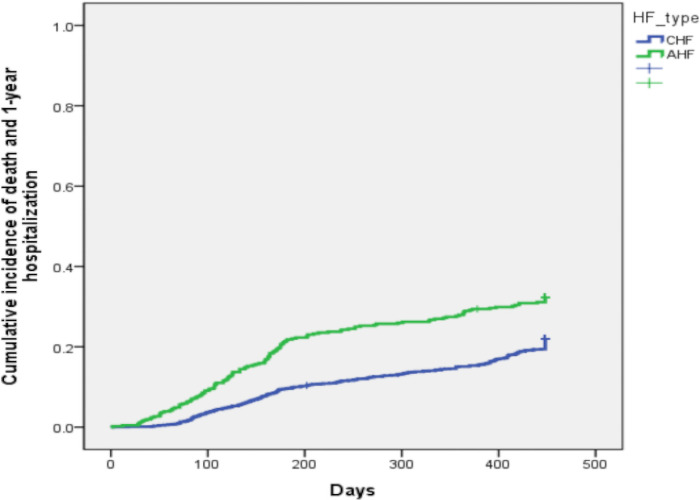
Cumulative incidence plots of death and heart failure (HF) hospitalization in acute heart failure and chronic heart failure patients.

The Fig 1 shows the Kaplan–Meier curves for all-cause mortality in AHF and in CHF patients. At three months, cumulative survial was 98% in CHF and 93% in CHF. At one year, cumulative survial was 90,5% in CHF and 78,4% in AHF. The Fig 2 shows the Kaplan-Meier curves for the combined event of all-cause mortality or hospitalization for HF. In CHF, cumulative events were 2.9% at 3 months and 15.1% at one year. Cumulative events were 7.8% at 3 months and 28.9% at one year in AHF.

The mortality rate during the initial hospitalization event for AHF was 7.4% (30 out of 408). The all-cause 1-year mortality rate was 22.8% among AHF patients and 10.6% among CHF patients «Table 2».

**Table 2:** Causes of death and rehospitalization of all study patients.

	Total population (n = 2040)	Acute HF (n = 408)	Chronic HF (n = 1632)	p-value
All-cause Mortality (%)	13	22.8	10.6	0.000
In-Hospital mortality (%)	3.3	7.4	1.9	0.000
12-months mortality and rehospitalization (%)	18.7	26.5	16.8	0.000
12-months rehospitalization (%)	7.3	5.9	7.7	0.21

The multivariate analysis showed older age, diabetes, heart rate, QRS duration, anemia, residual congestion and the absence of angiotensin-converting enzyme inhibitors (ACEI)/angiotensin receptor antagonists (ARBs) treatment to be independent predictors of 1-year all-cause mortality in CHF patients «Table 3».

**Table 3:** Predictors of all-cause of 1- year mortality in CHF patients (multivariable analysis).

	HR (95% CI)	p-value
**Age ≥ 75 years (yes vs no)**	1.894 (1.256–2.856)	0.002
**Diabetes (yes vs no)**	1.520 (1.047–2.206)	0.028
**Heart rate ≥ 70 bpm (yes vs no)**	1.731 (1.046–2867)	0.033
**QRS duration > 150 msec (yes vs no)**	2.641 (1.279–5.455)	0.009
**Anemia (yes vs no)**	1.887 (1.081–3.293)	0.025
**ACEI/ ARBs treatment (yes vs no)**	0.466 (0.309–0.703)	0.000
**Loop diuretics optimization (yes vs no)**	0.595 (0.405–0.874)	0.008

The female sex, New York Heart Association (NYHA) class III or IV and the absence of ACEI/ARBs treatment were independent predictors of 1-year all-cause mortality in AHF patients «Table 4».

**Table 4:** Predictors of all-cause of 1- year mortality in AHF patients (multivariable analysis).

	HR (95% CI)	p-value
**Female sex (yes vs no)**	2.004 (1.129–3.555)	0.017
**NYHA III—IV (yes vs no)**	2.525 (1.316–4.867)	0.005
**ACEI/ ARBs treatment (yes vs no)**	0.449 (0.252–0.798)	0.006

Among CHF patients, the older age, diabetes, anemia, reduced EF, ischemic etiology, residual congestion and the absence of ACEI/ ARBs treatment were independent predictors of 1-year cumulative rates of rehospitalization and mortality «Table 5».

**Table 5:** Predictors of all-cause of 1-year rehospitalization and mortality in CHF patients (multivariable analysis).

	HR (95% CI)	p-value
**Age ≥ 75 years (yes vs no)**	1.995 (1.452–2.741)	0.000
**Diabetes (yes vs no)**	1.645 (1.242–2.178)	0.001
**Anemia (yes vs no)**	1.749 (1.109–2.759)	0.016
**LV EF < 40% (yes vs no)**	1.327 (1.006–1.750)	0.045
**Ischemic etiology (yes vs no)**	1.348 (1.010–1.798)	0.042
**ACE/ ARBs treatment (yes vs no)**	0.553 (0.399–0.767)	0.000
**Loop diuretics optimization (yes vs no)**	0.490 (0.369–0.650)	0.000

The female sex and the New York Heart Association (NYHA) class III or IV were independent predictors of 1-year all-cause mortality and rehospitalization in AHF patients «Table 6».

**Table 6:** Predictors of all-cause of 1- year mortality and rehospitalization in AHF patients (multivariable analysis).

	HR (95% CI)	p-value
**Female sex (yes vs no)**	1.996 (1.188–3.355)	0.009
**NYHA III—IV (yes vs no)**	2.629 (1.514–4.565)	0.001

### Medications of HF-patients

During the out-patient visit, both ACEI/ ARBs and beta-blockers were the most prescribed medications (67.8% and 65.1% respectively). The medication prescribed for CHF patients at baseline and 1-year follow-up are presented in «Table 7». The percentage of ACEI/ARBs, beta-blockers and mineralocorticoid receptor antagonists (MRAs) increased slightly from 68.9% to 73.2%, 67% to 71.9% and 28.8% to 31.3% respectively. Diuretics prescription fell from 52.2% to 23.0%. Prescription of ivabradine and sacubitril-valsartan remained stable during the follow-up.

**Table 7:** Pharmacological treatment of CHF patients during outpatient visit and at 1-year.

	Out-patient visit	At 1-year	P
ACEI/ ARBs (%)	68.9	73.2	0.000
Beta-blockers (%)	67	71.9	0.000
Aldosterone blockers (%)	28.8	31.3	0.000
Loop diuretics (%)	52.2	23	0.000
Ivabradine (%)	0.4	0.6	0.5
Sacubitril-valsartan (%)	0.2	0.2	1
Digitalis (%)	4.8	5.5	0.001

Table 8 summarizes the medications prescribed for AHF patients at discharge and at 1-year follow-up. Prescription of ACEI/ARBs, beta-blockers, MRAs rose significantly from 62.0% to 72.9%, 57.4% to 68.2% and 31.9% to 39.7% respectively. However, the use of diuretics fell from 65.9% to 26.9%. There were no statistically significant differences between baseline and 1-year follow-up prescription of ivabradine, digitalis and sacubitril-valsartan.

**Table 8:** Pharmacological treatment of AHF patients during discharge and at 1-year.

	Out-patient visit	At 1-year	p
ACEI/ ARBs (%)	62	72.9	0.000
Beta-blockers (%)	57.4	68.2	0.000
Aldosterone blockers (%)	31.9	39.7	0.000
Loop diuretics (%)	65.9	26.9	0.000
Ivabradine (%)	1.2	1.3	1
Sacubitril-valsartan (%)	0	0	1
Digitalis (%)	5.1	6.3	0.25

The use of implantable cardioverter defibrillators (ICDs), cardiac resynchronization therapy with defibrillation (CRT-D) and CRT-pacemaker (CRT-P) were used by 2.5%, 0.8% and 3.1%.

At 1-year follow-up, only 22.7% had optimal treatment. The ACEI/ARBs optimal doses were reached in 30.4% and those of beta-blockers in 40%.

The Nature-HF registry analyzed HF patients who were treated at the same hospitals by the same physicians and who had been followed up for 1 year. Of these patients, 408 (20%) were hospitalized with a diagnosis of AHF and 1632 (80%) were outpatients with CHF.

The main findings of this study are: (**i**) the all-cause 1-year mortality rates were 13% for all HF patients, 22.8% for AHF and 10.6% for CHF; (**ii**) the 1-year rates of hospitalization because of HF were 7.3% for all HF patients, 5.9% for AHF and 7.7% for CHF; (**iii**) the 1-year incidence rates of the combined event ‘all-cause mortality or HF hospitalization’ were 18.7% for all HF patients, 26.5% for AHF and 16.8% for CHF. Except the younger population age, the clinical characteristics were similar to those of previous studies [5, 6]. The incidence of the combined event of death or HF rehospitalization are lower in the present study, with 18.7% compared with 35.8% in the Pilot Survey [5] and 40.1% in the ESC-HF long term registry [7].

In this study, the rates of mortality and rehospitalization of CHF patients were significantly lower than that of AHF patients. This is may be due to more comorbidities especially diabetes (41.2% vs 34.5%, p = 0.01) and COPD (10.5% vs 5.8%, p = 0.001), atrial fibrillation (21.3% vs 14.5%, p = 0.000) and systolic LV dysfunction (53.9% vs 44.9%, p = 0.001) in AHF patients.

Among CHF patients, the rates of 1-year all-cause mortality and hospitalization were 10.6% and 7.7%. There is reduction in these rates compared with those of the north African population in the ESC-HF long term registry, in which rates were 15.6% and 10.1% respectively [7].

All-cause mortality among AHF patients is high, with a rate of in hospital death of 7.4% and 22.8% within 1 year. These rates are higher than in the ESC-HF Pilot Survey [5], in which the 1-year all-cause mortality rates were 17.4%, nearly equal to those of the ESC-HF long term registry [7], in which 1-year all-cause mortality rates were 23.6% and fewer than those of the north African population of the ESC-HF long term registry [7], in which rates were 29.1%.

The predictors of all-cause mortality among CHF patients in this study were similar to those found in previous studies: older age, accelerated heart rate, QRS duration, diabetes, anemia, congestion and failure to optimize treatment [7–11]. The predictors of all-cause mortality among AHF patients in this study were consistent with those observed in previous studies, in which mainly pulmonary congestion was predictive of an adverse outcome [12–14]. However, the female sex was also an independent predictor of worse prognosis in AHF. It seems to be a main particularity of HF north-African population. In fact, in the GREAT registry, women with AHF have a lower 1-year mortality unless less evidenced-based treatment than men [15]. In the Swede HF registry, after adjustments for mortality and morbidity, females had a better prognosis across the EF spectrum [6]. This finding may be explained by a delayed diagnosis or by the excess social stressors and less HF guideline-recommended treatments in females in our population. So that, we must improve management of AHF in women in Tunisia and north-African countries.

These results reinforce the recommendations that all HF patients should not be discharged until the signs of congestion have completely disappeared applying recommended treatment protocols [16]. The congestion must be treated in CHF patients as well in AHF ones and the HF guideline-recommended treatments must be initiated before discharge. The lower death and cumulative rehospitalization rates among both AHF and CHF patients may be attributable in part to the younger age and the improvement in the management of HF patients after current HF guidelines consisting with more frequent prescription of beta-blockers and ACEI/ARBs [2, 4]. However, the prescription of ivabradine and sacubitril-valsartan was still limited. This is due to probably to the cost and the absence of the reimbursement of ivabradine and the unavailability and the absence of local authorization of sacubitril-valsartan in Tunisia during the study period. In addition, the use of implantable devices (ICDs and CRTs) was still infrequent. As an example, the ICDs implantation ranges from 2.5% in our patients to 21.3% in Western Europe [7]. This is also possibly due to multifactorial causes involving essentially cost, and reimbursement systems in our country [17]. The treatment optimization is still also limited. At 1-year follow-up, only 22.7% had optimal treatment. The ACEI/ARBs optimal doses were reached in 30.4% and those of beta-blockers in 40%. These rates were comparable to those of previous registries [18, 19].

Finally, it seems important to integrate HF disease management programs, especially those with nursing staff involvement in order to ameliorate the HF prognosis and reduce rate of hospitalization for HF [20–23].

### Study limitations

This study has some important limitations. First of all, although the criteria for HF are well established in the guidelines [2, 4], patients included in the NATURE-HF registry are diagnosed by local physicians and are not validated centrally. Second, the patients included are only enrolled among those admitted to the cardiology department or seen in outpatient cardiology consultations. The HF patients seen in other departments, such as the emergency unit, were therefore not taken into account.

## Conclusions

In comparison with the results of previous studies, the results of the present study, show that 1-year mortality is reduced but is still important among HF patients. The reduction is particularly seen in CHF patients. This is possibly due to increasing prescription of HF guideline-recommended treatment. However, full adherence to current guidelines is not totally achieved, due to in part to logistical and cost issues. Among AHF patients, females had also worse prognosis. This finding deserves attention considering differences with previous studies. The NATURE-HF registry provides important data to improve the management and outcomes of HF patients in north-African countries.

## Supporting information

S1 File TREND statement checklist. (PDF)

S1 Table Baseline characteristics of patients with chronic heart failure (N = 1632) according to left ventricle ejection fraction EF: Ejection fraction; * Kruskal-Wallis test. (PDF)

S2 Table Baseline characteristics of patients with acute heart failure (N = 408) according to EF: Ejection fraction; * Kruskal-Wallis test EF: Ejection fraction; * Kruskal-Wallis test. (PDF)

S3 Table Medications of the chronic heart failure during out-patients visit according to the left ventricle ejection fraction EF: Ejection fraction. (PDF)

S4 Table Medications of the acute heart failure at discharge according to the left ventricle ejection fraction EF: Ejection fraction. (PDF)
