# Correction: Epidemiology of heart failure and long-term follow-up outcomes in a north-African population: Results from the NAtional TUnisian REgistry of Heart Failure (NATURE-HF)

**DOI:** 10.1371/journal.pone.0335651

**Published:** 2025-10-29

**Authors:** Leila Abid, Salma Charfeddine, Ikram Kammoun, Manel Ben Halima, Hedi Ben Slima, Meriem Drissa, Khadija Mzoughi, Dorra Mbarek, Leila Riahi, Saoussen Antit, Afef Ben Halima, Wejdene Ouechtati, Emna Allouche, Mehdi Mechri, Chedi Youssfi, Ali Khorchani, Kais Sammoud, Khaled Zaouia, Rami Tlili, Sana Ouali, Faten Triki, Sonia Hamdi, Selim Boudich, Marwa Chebbi, Mouna Hentati, Amani Farah, Habib Triki, H. Ghardallou, H. Radoui, Sofien Zayed, F. Azaiez, Fadoua Omri, Akram Zouari, Zine Ben Ali, A. Najjar, Houssem Thabet, Mouna Chaker, Samar Mohammed, Abdelhamid Ben Jmaa, Haithem Tangour, Yassine Kammoun, Mahmoud Cheikh Bouhlel, S. Azeiz, R. Gtaief, S. Mashki, Aymen Amri, Hela Naanea, Raoudha Othmani, Iheb Chahbani, Houcine Zargouni, Syrine Abid, Mokded Ayari, Ines Ben Ameur, Ali Guesmi, Nejeh Ben Halima, Habib Haouala, Wafa Fehri, Essia Boughzela, Lilia Zakhama, Soraya Ben Youssef, Wided Nasraoui, Rachid Boujneh, Nedia Barakett, Sondos Kraiem, Hbiba Drissa, Ali Ben Khalfalah, Habib Gamra, Salem Kachboura, Yosra Majdoub, Elifa Kanoun, Faiez Zannad, Sami Milouchi, Alexandre Mebaza, Samir Kammoun, Sami Mourali, Karima Hezbri, Faouzi Addad

There are number of errors in Tables 1–8. Please see the complete, correct Tables 1–8 here.

In the Abstract, there is an error in the fourth sentence. The correct sentence is: Of these, 1632 (80%) were outpatients with chronic HF (CHF). The mean hospital stay for hospitalized patients was 8.7 ± 8.2 days. There’s another error in the seventh and eight sentence. The correct sentence are: Among CHF patients, the older age, diabetes, anemia, reduced EF, ischemic etiology, residual congestion and the absence of ACEI/ ARBs treatment were independent predictors of 1-year combined rates of HF rehospitalization and mortality. Female sex and functional status were independent predictors of 1-year all-cause mortality and rehospitalization in AHF patients.

In the Introduction, there is an error in the fourth sentence. The correct sentence is: In Tunisia and north-african countries, HF is a public health problem considering its current frequency and this is mainly linked to an aging of the Tunisian population and an increase in coronary and hypertensive patients (5).

In the Study design and patient’s enrollment subsection of the Materials and methods, there are errors in the first and second paragraph. The correct paragraphs are: The NATURE- HF registry was a national, Tunisian, observational, longitudinal, prospective and multicentric registry carried out on a follow-up period of 13 months: 01 month of patients’ recruitment and 12 months of follow-up. The protocol of the NATURE HF registry has been approved by the Tunisian Society of Cardiology and Cardiovascular Surgery. The NATURE HF study has been submitted to ClinicalTrials.gov and registered under the identifier NCT03262675. An ethical approval letter has been obtained from the ethic committee of the Abderrahmen Mami Pneumology and Phthisiology Hospital. A written informed consent was obtained before the inclusion in the study. The data were collected after giving detailed information about the study to the patient. We included all outpatients with chronic HF (CHF) and those hospitalized for acute HF (AHF) de novo or not. Any violation of the study protocol was exposed to the Steering Committee which would decide on the exclusion of the patient in question before final recruitment. The selection of patients eligible for inclusion and non-inclusion criteria was made at the cardiology consultation level or during cardiology or emergencies hospitalizations. A total of 250 Cardiologists (public sector and liberal sector) participated in the patients’ enrollment. Patient inclusion occurred consecutively until the end of the inclusion period. The inclusion began on October 02, 2017 for a duration of 01 months. A Regular follow-up was done at 12 months after inclusion. Given the observational nature of the NATURE-HF study, no specific treatment or intervention is was planned in the management of HF. Patients were cared according to the usual medical habits.

The diagnosis of HF was at the discretion of investigator according to the current guidelines (2,4). Patients had ECG recordings and echocardiogram at the time of enrollment. Echocardiograms were performed for all patients at the time of inclusion. The main exclusion criteria were an estimated life expectancy <12 months for extra-cardiac pathology, isolated right heart failure, pregnancy, end-stage or severe renal insufficiency with creatinine clearance <15ml/ min, hemodialysis patient, cardiac surgery scheduled within 3 months and congenital heart disease.

In the Statistical analysis subsection of the Materials and methods, there are errors in the first and second paragraph. The correct paragraphs are: Categorical variables were expressed as frequencies and percentages. For quantitative variables, we checked the normality of the distribution by the Kolmogorov-Smirnov test and the Shapiro-Wilk test. An estimate of the means with their standard deviations and of the median with interquartile range (IQR), was thus carried out. The baseline characteristics and type of treatments were also reported. The plots of Kaplan–Meier curves for time to all-cause death and time to all-cause death or HF hospitalization were performed. The associations between the variables were studied using hypothesis tests. The comparison between two categorical variables was carried out by the Pearson “chi2” test. When more than 20% of cells have expected frequencies < 5, the Fisher’s exact test was used. The Student test was used for the comparison of two means when the distribution is Gaussian and by the non-parametric U test of Mann-Whitney or Kruskal-Wallis when the distribution is not Gaussian. In addition, the study population were divided into outpatients with CHF and inpatients with AHF. The McNemar test was used to determine if there are differences in paired data during the follow-up in the different study groups. Plots of cumulative incidence of HF hospitalization considering competing risks of death in the two groups were presented.

All variables that were statistically significant at univariate analysis (Log-rank Kaplan-Meier), and those considered of relevant clinical interest with a risk of error of 20% were included in a multivariable model (Cox model) to identify the independent predictors with adjusted Odds Ratio (aOR) of all-cause death, death and rehospitalization/ HF from the study entry to 1-year follow-up, separately for AHF and CHF. Age, systolic blood pressure and ejection fraction (EF) were considered as continuous variables while the remaining were considered as categorical variables. A P-value <0.05 was considered statistically significant.

In the Results and discussion, there are errors in the paragraph. The correct paragraph is: A total of 2040 patients were included in the study. Of these, 408 (20%) were inpatients hospitalized with a diagnosis of acute HF (AHF) and 1632 (80%) were outpatients with chronic HF (CHF). For hospitalized HF patients, the mean hospital stay was 8.7 ± 8.2 days [2; 55]. At 1 year, 32 AHF patients (7.8%) and 68 CHF patients (4.1%) had been lost to follow-up.

In the Baseline characteristics subsection of the Results and discussion, the sixth sentence is missing from the first paragraph. The missing sentence is: Only 2.7% of all patients had brain natriuretic peptides (BNP) levels at inclusion. The mean BNP level was 332.9 ± 267.2 pg/mL.

In the Baseline characteristics subsection of the Results and discussion, the second to fourth paragraphs are missing. The missing paragraphs are: In the total HF population, females were older than males (65.69 ± 12.9 vs. 62.69 ± 12.4, p < 10^−3^). The female patients had more diabetes (40.9% vs. 33.8%, p = 0.02), hypertension (51.9% vs. 35.7%, p < 10^−3^), atrial fibrillation (23.9% vs. 12.7%, p < 10^−3^), anemia (11.6% vs. 6.2%, p < 10^−3^) and renal failure (46.7% vs. 34.9%, p = 0.003).

Patients with CHF and preserved EF were more female and had more hypertension, diabetes, atrial fibrillation than those with reduced and mid-range EF (p < 10^−3^, 0.002, 0.002 and <10^−3^ respectively) (S1 Table).

Patients with AHF and preserved EF were more female, older and had more hypertension and atrial fibrillation than those with reduced and mid-range EF (p 0.02, 0.01, 0.005 and 0.001 respectively) (S2 table).

In the Follow-up subsection of the Results and discussion, there are errors in the first paragraph. The correct paragraph is: The Fig 1 shows the Kaplan–Meier curves for all-cause mortality in AHF and in CHF patients. At three months, cumulative survial was 98% in CHF and 93% in AHF. At one-year, cumulative survival was 90.5% in CHF and 78.4% in AHF. The Fig 2 shows the Kaplan-Meier curves for the combined event of all-cause mortality or hospitalization for HF. In CHF, composite events were 2.9% at 3 months and 15.1% at one year. In AHF, composite events were 7.8% at 3 months and 28.9% at one year.

In the Follow-up subsection of the Results and discussion, there are errors in the fifth paragraph. The correct paragraph is: Among CHF patients, the older age, diabetes, anemia, reduced EF, ischemic etiology, residual congestion and the absence of ACEI/ ARBs treatment were independent predictors of 1-year composite events of rehospitalization and mortality (Table 5).

In the Medications of HF-patients subsection of the Results and discussion, there are errors in the first paragraph. The correct paragraph is: During the out-patient visit in CHF, both ACEI/ ARBs and beta-blockers were the most prescribed medications (68.9% and 67% respectively). Patients with CHF and preserved EF had less prescribed ACEI/ARBs, beta-blockers and mineralocorticoid receptor antagonists (MRAs) than those with reduced and mid-range EF (S3 Table). The medication prescribed for CHF patients at baseline and 1-year follow-up are presented in **Table 7**. The percentage of ACEI/ARBs, beta-blockers and MRAs increased slightly from 68.9% to 83.8%, 67% to 82.5% and 28.8% to 35.9% respectively. Diuretics prescription fell from 52.2% to 26.4%. Prescription of ivabradine and sacubitril-valsartan remained stable during the follow-up.

In the Medications of HF-patients subsection of the Results and discussion, a paragraph is missing after the first paragraph. The missing paragraph is: At discharge, patients with AHF and preserved or mid-range EF had more prescribed ACEI/ARBs, beta-blockers, loop diuretics and digoxin than those with reduced EF (S4 Table).

In the Medications of HF-patients subsection of the Results and discussion, there is an error in the first sentence of the fourth paragraph. The correct sentence is: At 1-year follow-up, only 22.7% had optimal treatment defined as the presence of the 3 recommended medications classes at the maximally-tolerated doses.

In the Medications of HF-patients subsection of the Results and discussion, a paragraph is missing after the ninth paragraph. The missing paragraph is: The outcomes rates reduction compared with the ESC-HF cohort especially in CHF patients may be due to the exclusion of severe renal patients, patients on hemodialysis, patients with congenital heart disease and those with planned cardiac surgery within 3 months in the NATURE-HF registry.

In the Medications of HF-patients subsection of the Results and discussion, there are errors in the tenth paragraph. The correct paragraph is: The predictors of all-cause mortality among CHF patients in this study were similar to those found in previous studies: older age, QRS duration, diabetes, anemia, congestion,failure to optimize treatment and LV dysfunction (9),(10),(11), (12), (13). As reported previously, the LVEF <40% was an independent predictor of composite events (9). The predictors of all-cause mortality among AHF patients in this study were consistent with those observed in previous studies, in which mainly pulmonary congestion was predictive of an adverse outcome (14), (15), (16). However, in this study, the female sex was an independent predictor of worse prognosis in AHF. This finding seems to be a main particularity of north-African patients with HF. In fact, in the GREAT registry, women with AHF have a lower 1-year mortality than men unless less evidenced-based treatment (17). In the Swedish HF registry, after adjustments with different predictors of all-cause mortality and HF hospitalization -such as age, HF duration, comorbidities, BMI, NYHA, BP, HR, HF etiology-, females had a better prognosis across the EF spectrum (7). This finding may be explained by a delayed diagnosis or by the excess social stressors and less HF guideline-recommended treatments in females in our population. Females were also older and had more co-morbidities (diabetes, hypertension, atrial fibrillation, anemia and renal dysfunction). So that, we must improve management of HF in women in Tunisia and north-African countries.

In the Medications of HF-patients subsection of the Results and discussion, there are sentences missing in the eleventh paragraph. The correct paragraph is: These results reinforce the recommendations that all HF patients should not be discharged until the signs of congestion have completely disappeared applying recommended treatment protocols (18). The congestion must be treated in CHF patients as well in AHF ones and the HF guideline-recommended treatments must be initiated before discharge. The lower death and cumulative rehospitalization rates among both AHF and CHF patients may be attributable in part to the younger age and the improvement in the management of HF patients after current HF guidelines consisting with more frequent prescription of beta-blockers and ACEI/ARBs (2),(4). However, the prescription of ivabradine and sacubitril-valsartan was still limited. This is due to probably to the cost and the absence of the reimbursement of ivabradine and the unavailability and the absence of local authorization of sacubitril-valsartan in Tunisia during the study period.

In this registry, unless patients with CHF and preserved EF had more comorbidities and less prescribed ACEI/ARBs, beta-blockers and MRAs, their prognosis was better than those with reduced and mid-range EF. The management of patients with HF preserved EF remains an on-going challenge and options to improve patients’ symptoms and quality of life include control of fluid overload, heart rate, risk factors, and comorbidities (19).

In addition, the use of implantable devices (ICDs and CRTs) was still infrequent. As an example, the ICDs implantation ranges from 2.5% in our patients to 21.3% in Western Europe (9). This is also possibly due to multifactorial causes involving essentially cost, and reimbursement systems in our country (20). The treatment optimization is still also limited. At 1-year follow-up, only 22.7% had optimal treatment. The ACEI/ARBs optimal doses were reached in 30.4% and those of beta-blockers in 40%. These rates were comparable to those of previous registries (21), (22).

In Study limitations subsection of the Results and discussion, the fifth and sixth sentence are missing. The missing sentences are: Finally, despite the well-established role of natriuretic peptides in the diagnosis and the severity assessment of HF, these markers were not available routinely in the different Tunisian centers. The absence of such objective criteria of HF and its severity is considered as a main limitation in this real-life registry.

**Table 1:** Baseline characteristics of the study population.

	Total population (n = 2040)	Acute HF (n = 408)	Chronic HF (n = 1632)	p-value
Age (years)				
Mean ± SD	63.56 ± 12.6	63.59 ± 12.9	63.54 ± 12.5	0.89^*^
Median [IQR]	64 [19–97]	64 [24–91]	64 [19–97]	
≥ 75 years (n, %)	421 (20.7%)	98 (24%)	324 (19.8%)	0.06
Females (n, %)	594 (29.2%)	117 (28.7%)	477 (29.3%)	0.85
Education (n, %)				
Illiterate	591 (29.1%)	123 (35.1%)	468 (31.8%)	0.50
Compulsory school (6–12 years)	698 (34.2%)	125 (35.7%)	573 (38.9%)	
Secondary school (13–18 years)	429 (21.5%)	79 (22.6%)	350 (23.7%)	
University (> 18 years)	106 (5.2%)	23 (6.6%)	83 (5.6%)	
Diabetes (n, %)	731 (35.8%)	168 (41.2%)	563 (34.5%)	0.01
Hypertension (n, %)	824 (40.4%)	172 (42.2%)	652 (40%)	0.42
Smoking (n, %)	508 (26.2%)	88 (21.7%)	420 (25.7%)	0.11
COPD (n, %)	138 (6.8%)	43 (10.5%)	95 (5.8%)	0.001
Coronary heart disease (n, %)	943 (46.2%)	170 (41.7%)	773 (47.4%)	0.03
NYHA II (n, %)	1367 (67.1%)	81 (19.8%)	1286 (78.9%)	<10^−3^
NYHA III (n, %)	562 (27.5%)	216 (52.9%)	346 (21.1%)	
NYHA IV (n, %)	111 (5.4%)	111 (27.2%)	0	
SBP (mmHg)				
Mean ± SD	123.37 ± 24.8	122.28 ± 29.0	123.95 ± 23.8	0.42^*^
Median [IQR]	120 [90–240]	120 [100–240]	120 [90–230]	
≤ 110 mmHg (n, %)	9 (0.5%)	2 (0.6%)	7 (0.5%)	0.68^**^
Heart rate (bpm)				
Mean ± SD	80.17 ± 17.5	88.10 ± 22.3	78.24 ± 15.6	<10^−3^^*^
Median [IQR]	77 [30–160]	84 [39–152]	75 [30–161]	
≥ 70 bpm (n, %)	1277 (77.1%)	275 (85.1%)	1004 (75.2%)	<10^−3^
ECG abnormalities				
Atrial fibrillation (n, %)	324 (15.9%)	87 (21.3%)	238 (14.5%)	<10^−3^
LBBB (n, %)	184 (9%)	41 (10%)	143 (8.8%)	
QRS duration				
>150 msec (n, %)	78 (3.8%)	22 (5.4%)	56 (3.4%)	0.002
Ejection fraction				
Mean ± SD	38.42 ± 10.5	36.96 ± 11.4	38.73 ± 10.2	0.005^*^
Median [IQR]	40 [15–63]	37 [12–65]	40 [14–87]	
< 40% (n, %)	1153 (56.5%)	265 (65%)	888 (54.4%)	0.001
40–50% (n, %)	760 (37.2%)	113 (27.7%)	647 (39.6%)	
>50% (n, %)	127 (6.2%)	30 (7.3%)	97 (6%)	
Mitral regurgitation (n, %)	463 (22.7%)	100 (24.5%)	363 (22.3%)	0.39
Renal dysfunction (n, %)	278 (38.4%)	60 (46.5%)	218 (36.6%)	0.03
Anemia (n, %)	158 (7.7%)	55 (13.5%)	103 (6.3%)	<10^−3^

* Mann Whitney test;

** Fisher test

**Table 2:** Causes of death and heart failure rehospitalization of all study patients.

	Total population (n = 2040)	Acute HF (n = 408)	Chronic HF (n = 1632)	p-value
12-month all-cause mortality (n, %)	265 (13%)	93 (22.8%)	172 (10.6%)	<10^−3^
In-Hospital mortality (n, %)	68 (3.3%)	30 (7.4%)	31 (1.9%)	<10^−3^
12-months mortality and rehospitalization/HF(n, %)	381 (18.7%)	108 (26.5%)	273 (16.8%)	0.01
12-months rehospitalization/HF (n, %)	149 (7.3%)	24 (5.9%)	125 (7.7%)	0.21

**Table 3:** Predictors of all-cause of 1- year mortality in CHF patients (multivariable analysis).

	aOR (95% CI)	p-value
**Age ≥ 75 years (yes vs no)**	1.652 (1.119–2.439)	0.012
**Diabetes (yes vs no)**	1.546 (1.083–2.208)	0.017
**Dyspnea NYHA III – IV**	1.502 (1.033–2.184)	0.033
**QRS duration > 150 msec (yes vs no)**	2.254 (1.167–4.352)	0.015
**Anemia (yes vs no)**	1.731 (1.037–2.889)	0.036
**ACEI/ ARBs treatment (yes vs no)**	0.513 (0.349–0.753)	0.001
Loop diuretics optimization^*^ **(yes vs no)**	0.577 (0.393–0.846)	0.005

aOR: adjusted Odds Ratio, CI: Confidence Interval.

* Loop diuretics optimization was defined as reduction to more or equal to the half initial dose

**Table 4:** Predictors of all-cause of 1- year mortality in AHF patients (multivariable analysis).

	aOR (95% CI)	p-value
**Female sex (yes vs no)**	1.877 (1.164–3.025)	0.010
**NYHA III – IV (yes vs no)**	2.359 (1.313–4.239)	0.004
**ACEI/ ARBs treatment (yes vs no)**	0.469 (0.291–0.756)	0.002

aOR: adjusted Odds Ratio, CI: Confidence Interval.

**Table 5:** Predictors of all-cause of 1-year rehospitalization and mortality in CHF patients (multivariable analysis).

	aOR (95% CI)	p-value
**University educational level (yes vs no)**	0.497 (0.259–0.956)	0.036
**Diabetes (yes vs no)**	1.493 (1.152–1.936)	0.002
**Anemia (yes vs no)**	1.620 (1.087–2.413)	0.018
**LV EF < 40% (yes vs no)**	1.402 (1.086–1.810)	0.010
**Ischemic etiology (yes vs no)**	1.359 (1.038–1.779)	0.026
**ACE/ ARBs treatment (yes vs no)**	0.534 (0.400–0.713)	<10^−3^
Loop diuretics optimization^*^ **(yes vs no)**	0.488 (0.374–0.638)	<10^−3^

aOR: adjusted Odds Ratio, CI: Confidence Interval.

* Loop diuretics optimization was defined as reduction to more or equal to the half initial dose

**Table 6:** Predictors of all-cause of 1- year mortality and rehospitalization in AHF patients (multivariable analysis).

	aOR (95% CI)	p-value
**Female sex (yes vs no)**	1.810 (1.220–2.685)	0.003
**NYHA III – IV (yes vs no)**	2.298 (1.445–3.656)	<10^−3^

aOR: adjusted Odds Ratio, CI: Confidence Interval.

**Table 7:** Pharmacological treatment of CHF patients during outpatient visits and at 1-year.

	Out-patient visit	At 1-year	p-value
ACEI/ ARBs (n, %)	1125 (68.9%)	1144 (83.8%)	<10^−3^
Beta-blockers (n, %)	1094 (67%)	1124 (82.5%)	<10^−3^
Aldosterone blockers (n, %)	470 (28.8%)	489 (35.9%)	<10^−3^
Loop diuretics (n, %)	852 (52.2%)	359 (26.4%)	<10^−3^
Ivabradine (n, %)	7 (0.4%)	9 (0.6%)	0.5
Sacubitril-valsartan (n, %)	3 (0.2%)	3 (0.2%)	1
Digitalis (n, %)	78 (4.8%)	86 (6.3%)	0.001

* The McNemar test for paired data

**Table 8:** Pharmacological treatment of AHF patients at discharge and at 1-year.

	At discharge	At 1-year	p-value
ACEI/ ARBs (n, %)	253 (62%)	253 (100%)	<10^−3^
Beta-blockers (n, %)	234 (57.4%)	250 (98.8%)	<10^−3^
Aldosterone blockers (n, %)	130 (31.9%)	149 (58.9%)	<10^−3^
Loop diuretics (n, %)	269 (65.9%)	101 (39.9%)	<10^−3^
Ivabradine (n, %)	5 (1.2%)	5 (1.9%)	1
Sacubitril-valsartan (n, %)	0	0	1
Digitalis (n, %)	21 (5.1%)	23 (9.1%)	0.25

* The McNemar test for paired data

## References

[1] Abid L, Charfeddine S, Kammoun I, Ben Halima M, Ben Slima H, Drissa M, et al. Epidemiology of heart failure and long-term follow-up outcomes in a north-African population: results from the national tunisian registry of heart failure (NATURE-HF). PLoS One. 2021;16(5):e0251658. doi: 10.1371/journal.pone.0251658 34014967 PMC8136726

