# Supplementary material for: Epidemiology of heart failure and long-term follow-up outcomes in a north-African population: Results from the NAtional TUnisian REgistry of Heart Failure (NATURE-HF)
Source: PLoS One. 2021 May 20;16(5):e0251658. doi: 10.1371/journal.pone.0251658 (PMC8136726; doi:10.1371/journal.pone.0251658)
Supplement: S1 Table — (PDF) [file pone.0251658.s002.pdf]

	Reduced EF (n= 888)	mid-range EF (n= 647)	Preserved EF (n= 97)	p-value
**Age (years)**				
**Mean ± SD**	63.53 ±12.3	63.41 ±12.6	64.81 ±14.5	0.58*
**Median [IQR]**	64 [19 – 97]	63 [25 – 93]	65 [21 – 95]	
**≥ 75 years (n, %)**	169 (19.1%)	130 (20.1%)	25 (25.8%)	0.28
**Female sex (n, %)**	224 (25.3%)	205 (31.7%)	48 (49.5%)	$<10^{-3}$
**Diabetes (n, %)**	273 (30.7%)	256 (39.6%)	34 (35.1%)	0.002
**Hypertension (n, %)**	321 (36.2%)	285 (44%)	46 (47.4%)	0.002
**Smoking (n, %)**	212 (29.4%)	188 (30.2%)	14 (15.9%)	$<10^{-3}$
**COPD (n, %)**	53 (6%)	34 (5.3%)	8 (8.2%)	0.48
**Coronary heart disease (n, %)**	380 (42.8%)	369 (57.0%)	24 (24.7%)	$<10^{-3}$
**NYHA III (n, %)**	219 (34.6%)	95 (16.1%)	32 (40%)	$<10^{-3}$
**SBP (mmHg)**				
**Mean ± SD**	121.69 ±23.1	125.07 ±23.4	127.27 ±29.5	$<10^{-3}$*
**Median [IQR]**	120 [90 – 220]	120 [100 – 200]	130 [120 – 230]	
**Heart rate (bpm)**				
**Mean ± SD**	79.08 ±15.5	76.88 ±15.1	81.67 ±17.9	0.003*
**Median [IQR]**	76 [48 – 155]	75 [30 – 161]	78 [50 – 160]	
**≥ 70 bpm (n, %)**	472 (75.3%)	449 (73.2%)	83 (87.4%)	0.01
**Atrial fibrillation (n, %)**	108 (17%)	96 (15.6%)	34 (35.8%)	$<10^{-3}$
**QRS duration >150 msec (n, %)**	40 (6.5%)	14 (2.3%)	2 (2.1%)	0.001
**Renal dysfunction (n, %)**	102 (35.9%)	101 (36.3%)	15 (45.5%)	0.55
**Anemia (n, %)**	44 (5%)	51 (7.9%)	8 (8.2%)	0.04

EF: ejection fraction; * Kruskal-Wallis test
