# Supplementary material for: Epidemiology of heart failure and long-term follow-up outcomes in a north-African population: Results from the NAtional TUnisian REgistry of Heart Failure (NATURE-HF)
Source: PLoS One. 2021 May 20;16(5):e0251658. doi: 10.1371/journal.pone.0251658 (PMC8136726; doi:10.1371/journal.pone.0251658)
Supplement: S2 Table — (PDF) [file pone.0251658.s003.pdf]

	Reduced EF (n= 265)	mid-range EF (n= 113)	Preserved EF (n= 30)	p-value
**Age (years)**				
**Mean ± SD**	64.20 ±13.1	61.15 ±12.4	67.37 ±12.4	0.01*
**Median [IQR]**	64 [24 – 91]	61 [24 – 86]	67.5 [38 – 85]	
**≥ 75 years (n, %)**	65 (24.5%)	21 (18.6%)	12 (40%)	0.04
**Female sex (n, %)**	69 (26%)	33 (29.2%)	15 (50%)	0.02
**Diabetes (n, %)**	103 (38.9%)	53 (46.9%)	12 (40%)	0.34
**Hypertension (n, %)**	103 (38.9%)	48 (42.5%)	21 (70%)	0.005
**Smoking (n, %)**	67 (29.8%)	14 (12.5%)	7 (23.3%)	$<10^{-3}$
**COPD (n, %)**	28 (10.6%)	12 (10.6%)	3 (10%)	0.99
**Coronary heart disease (n, %)**	106 (40%)	57 (50.4%)	7 (23.3%)	0.01
**NYHA III (n, %)**	146 (55.1%)	50 (41.2%)	20 (66.6%)	$<10^{-3}$
**SBP (mmHg)**				
**Mean ± SD**	118.91 ±25.8	124.36 ±31.7	135.10 ±33.3	$<10^{-3}$*
**Median [IQR]**	120 [110 – 240]	120 [100 – 240]	139.5 [130 – 180]	
**Heart rate (bpm)**				
**Mean ± SD**	93.10 ±21.5	81.37 ±20.1	82.67 ±27.6	$<10^{-3}$*
**Median [IQR]**	90 [45 – 150]	76 [39 – 152]	75 [40 – 140]	
**≥ 70 bpm (n, %)**	167 (63.0%)	89 (78.7%)	19 (63.3%)	$<10^{-3}$
**Atrial fibrillation (n, %)**	43 (16.2%)	27 (23.8%)	17 (56.7%)	0.001
**QRS duration >150 msec (n, %)**	14 (5.2%)	6 (5.3%)	2 (6.7%)	0.56
**Renal dysfunction (n, %)**	38 (14.3%)	14 (12.4%)	8 (26.6%)	0.11
**Anemia (n, %)**	29 (10.9%)	15 (13.3%)	11 (36.7%)	0.002

EF: ejection fraction; * Kruskal-Wallis test
