# Supplementary material for: Epidemiology of heart failure and long-term follow-up outcomes in a north-African population: Results from the NAtional TUnisian REgistry of Heart Failure (NATURE-HF)
Source: PLoS One. 2021 May 20;16(5):e0251658. doi: 10.1371/journal.pone.0251658 (PMC8136726; doi:10.1371/journal.pone.0251658)
Supplement: S3 Table — (PDF) [file pone.0251658.s004.pdf]

	**Reduced EF (n= 888)**	**Mid-range EF (n= 647)**	**Preserved EF (n= 97)**	**p-value**
**ACEI/ ARBs (n, %)**	527 (59.3%)	531 (82.1%)	67 (69.1%)	$<10^{-3}$
**Beta-blockers (n, %)**	535 (60.2%)	507 (78.4%)	52 (53.6%)	$<10^{-3}$
**Aldosterone blockers (n, %)**	325 (36.6%)	126 (19.5%)	19 (19.6%)	$<10^{-3}$
**Diuretics (n, %)**	491 (55.3%)	294 (45.4%)	67 (69.1%)	$<10^{-3}$
**Digoxin (n, %)**	38 (4.3%)	29 (4.5%)	11 (11.3%)	0.007

EF: ejection fraction
