# Supplementary material for: Epidemiology of heart failure and long-term follow-up outcomes in a north-African population: Results from the NAtional TUnisian REgistry of Heart Failure (NATURE-HF)
Source: PLoS One. 2021 May 20;16(5):e0251658. doi: 10.1371/journal.pone.0251658 (PMC8136726; doi:10.1371/journal.pone.0251658)
Supplement: S4 Table — (PDF) [file pone.0251658.s005.pdf]

	**Reduced EF (n= 265)**	**Mid-range EF (n= 113)**	**Preserved EF (n= 30)**	**p-value**
**ACEI/ ARBs (n, %)**	145 (54.7%)	87 (77%)	21 (70%)	$<10^{-3}$
**Beta-blockers (n, %)**	130 (49.1%)	86 (76.1%)	18 (60%)	$<10^{-3}$
**Aldosterone blockers (n, %)**	87 (32.8%)	32 (28.3%)	11 (36.7%)	0.58
**Diuretics (n, %)**	165 (62.3%)	78 (69%)	26 (86.7%)	0.02
**Digoxin (n, %)**	8 (3%)	8 (7.1%)	5 (16.7%)	0.003

EF: ejection fraction
